# CXCR3 Identifies Human Naive CD8^+^ T Cells with Enhanced Effector Differentiation Potential

**DOI:** 10.4049/jimmunol.1901072

**Published:** 2019-11-18

**Authors:** Gabriele De Simone, Emilia M. C. Mazza, Antonino Cassotta, Alexey N. Davydov, Mirela Kuka, Veronica Zanon, Federica De Paoli, Eloise Scamardella, Maria Metsger, Alessandra Roberto, Karolina Pilipow, Federico S. Colombo, Elena Tenedini, Enrico Tagliafico, Luca Gattinoni, Domenico Mavilio, Clelia Peano, David A. Price, Satya P. Singh, Joshua M. Farber, Valentina Serra, Francesco Cucca, Francesco Ferrari, Valeria Orrù, Edoardo Fiorillo, Matteo Iannacone, Dmitriy M. Chudakov, Federica Sallusto, Enrico Lugli

**Affiliations:** * Laboratory of Translational Immunology, Humanitas Clinical and Research Center, 20089 Rozzano, Milan, Italy;; † Institute for Research in Biomedicine, Faculty of Biomedical Sciences, USI, 6500 Bellinzona, Switzerland;; ‡ Institute of Microbiology, ETH Zurich, 8093 Zurich, Switzerland;; § Central European Institute of Technology, 621 00 Brno, Czech Republic;; ¶ Division of Immunology, Transplantation and Infectious Diseases and Experimental Imaging Center, IRCCS, San Raffaele Scientific Institute and Vita-Salute San Raffaele University, 20132 Milan, Italy;; ‖ Humanitas Flow Cytometry Core, Humanitas Clinical and Research Center, 20089 Rozzano, Milan, Italy;; # Department of Life Sciences, University of Modena and Reggio Emilia, 41125 Modena, Italy;; ** Center for Cancer Research, National Cancer Institute, Bethesda, MD 20892;; †† Regensburg Center for Interventional Immunology, University Regensburg and University Hospital Regensburg, 93053 Regensburg, Germany;; ‡‡ Unit of Clinical and Experimental Immunology, Humanitas Clinical and Research Center, 20089 Rozzano, Milan, Italy;; §§ Department of Medical Biotechnologies and Translational Medicine, University of Milan, 20122 Milan, Italy;; ¶¶ Division of Genetic and Biomedical Research, UoS Milan, National Research Council, 20089 Rozzano, Milan, Italy;; ‖‖ Genomic Unit, Humanitas Clinical and Research Center, 20089 Rozzano, Milan, Italy;; ## Division of Infection and Immunity, Cardiff University School of Medicine, Cardiff CF14 4XN, United Kingdom;; *** Systems Immunity Research Institute, Cardiff University School of Medicine, Cardiff CF14 4XN, United Kingdom;; ††† Laboratory of Molecular Immunology, National Institute of Allergy and Infectious Diseases, National Institutes of Health, Bethesda, MD 20892;; ‡‡‡ IRGB, National Research Council, 09042 Monserrato, Italy;; §§§ IFOM, FIRC Institute of Molecular Oncology, 20139 Milan, Italy;; ¶¶¶ Shemyakin and Ovchinnikov Institute of Bioorganic Chemistry, 117997 Moscow, Russia; and; ‖‖‖ Pirogov Russian National Research Medical University, 117997 Moscow, Russia

## Abstract

In mice, the ability of naive T (T_N_) cells to mount an effector response correlates with TCR sensitivity for self-derived Ags, which can be quantified indirectly by measuring surface expression levels of CD5. Equivalent findings have not been reported previously in humans. We identified two discrete subsets of human CD8^+^ T_N_ cells, defined by the absence or presence of the chemokine receptor CXCR3. The more abundant CXCR3^+^ T_N_ cell subset displayed an effector-like transcriptional profile and expressed TCRs with physicochemical characteristics indicative of enhanced interactions with peptide–HLA class I Ags. Moreover, CXCR3^+^ T_N_ cells frequently produced IL-2 and TNF in response to nonspecific activation directly ex vivo and differentiated readily into Ag-specific effector cells in vitro. Comparative analyses further revealed that human CXCR3^+^ T_N_ cells were transcriptionally equivalent to murine CXCR3^+^ T_N_ cells, which expressed high levels of CD5. These findings provide support for the notion that effector differentiation is shaped by heterogeneity in the preimmune repertoire of human CD8^+^ T cells.

## Key Points

- CXCR3 identifies human naive CD8^+^ T cells with biased effector potential.
- Human naive CD8^+^ T cell subsets are functionally and transcriptionally distinct.
- Effector potential correlates with the physicochemical attributes of expressed TCRs.

## Introduction

Mature naive T (T_N_) cells are released from the thymus with predetermined specificities encoded by the somatically rearranged TCR. The human T_N_ cell repertoire incorporates >10^8^ different TCRs (1, 2), and a single TCR can recognize >10^6^ different peptide Ags (3). This inherent cross-reactivity enables comprehensive recognition of exogenous Ags and ensures that T_N_ cells can also interact with self-derived Ags (4). In mice, TCR interactions with self-derived peptide–MHC class I (pMHCI) complexes generate tonic signals, which do not induce effector responses in the absence of inflammation but are required for the survival of CD8^+^ T_N_ cells in the periphery (5, 6). These signals also drive low-level homeostatic proliferation in conjunction with IL-7, which in turn maintains a diverse repertoire of clonotypically expressed TCRs in the CD8^+^ T_N_ cell pool, even under conditions of reduced thymic output (4, 6).

In response to immune activation, T_N_ cells differentiate into effector cells that migrate to peripheral tissues and eliminate the inciting Ag. Once this process is complete, small numbers of Ag-specific T cells survive and become long-lived memory T (T_MEM_) cells (7), which exhibit diverse epigenetic, functional, metabolic, and transcriptional properties (8–13). T_N_ cells have long been considered largely homogenous at the population level (11, 14–16). However, the recent application of emerging single-cell technologies has shown that individual clonotypes in the T_N_ cell pool can behave very differently in response to Ag recognition via the TCR. For example, single-cell adoptive transfer and barcoding experiments in mouse challenge models have demonstrated that some CD8^+^ T_N_ cells proliferate extensively and differentiate into effector cells, whereas other CD8^+^ T_N_ cells proliferate to a lesser extent and differentiate into memory cells (17, 18). Another report described similar heterogeneity in the murine CD4^+^ T_N_ cell pool and further suggested that individual cellular trajectories were determined primarily by Ag density and TCR dwell time (19). All of these studies concluded that classical T cell responses arise via population averaging rather than uniform behavior (17–19).

In mice, the ability of T_N_ cells to respond to exogenous Ags correlates with the level of cross-reactivity against self-derived Ags, which can be quantified via the surrogate marker CD5 (20–22). Functionally distinct subsets of murine T_N_ cells have also been identified on this basis. For example, CD8^+^ T_N_ cells that express high levels of CD5 are hyperresponsive to the homeostatic cytokines IL-2 and IL-7 (23) and upregulate genes associated with effector differentiation (22), and CD4^+^ T_N_ cells that express high levels of CD5 display enhanced signaling potency downstream of the TCR (20, 21). CD5 has been used as a proxy for similar purposes in phenotypic analyses of human CD8^+^ T_N_ cells (24, 25), However, it remains unclear whether such functional heterogeneity exists among human CD8^+^ T_N_ cells and, if so, to what extent it determines the efficacy of adaptive immune responses.

## Materials and Methods

### Study approvals

The use of human samples was approved by the relevant Institutional Review Boards. Ethical approval for the use of buffy coats was granted by the Humanitas Research Hospital and the Swiss Federal Office of Public Health (A000197/2). Ethical approval for the use of peripheral blood (PB) samples from the SardiNIA study was granted by the Consiglio Nazionale delle Ricerche (0078008/2017). Ethical approval for the use of lymph nodes (LNs) from patients with head and neck cancer was granted by the Humanitas Research Hospital (700/2010). Mouse protocols were approved by the Humanitas Institutional Animal Care and Use Committee and the Italian Ministry of Health (452/2018-PR).

### Cells

PBMCs were isolated from buffy coats via standard density gradient centrifugation. In most assays, PBMCs were used immediately after isolation. In some assays, PBMCs were used after cryopreservation at −80°C in FBS containing 10% DMSO. Naive CD8^+^ T cells were enriched by magnetic separation using a MojoSort Human CD8^+^ Naive T Cell Isolation Kit (BioLegend), and total CD8^+^ T cells were enriched by magnetic separation using an EasySep Human CD8^+^ T Cell Isolation Kit (Stemcell Technologies).

### Human tissue samples

LNs were surgically removed from patients with head and neck cancer (age, 31–69 y) and processed as described previously (26). Information on tissue samples from publicly available mass cytometry by time-of-flight (CyTOF) data reported in this study can be found in Supplemental Table II from Wong et al. (27).

### Flow cytometry and cell sorting

**FIGURE 1.**
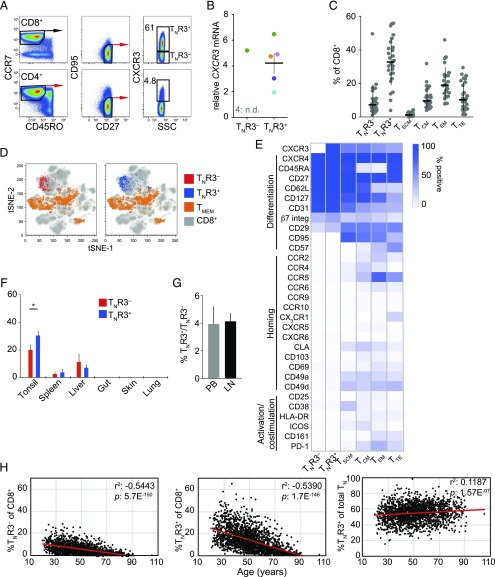
CXCR3 identifies two subsets of T_N_ cells in humans. (**A**) Representative flow cytometric analysis of CXCR3 expression on the surface of T_N_ cells (CD45RO^−^CCR7^+^CD27^+^CD95^−^). Numbers indicate the percentage of cells in each gate. (**B**) Expression of *CXCR3* relative to *B2M* mRNA in flow-sorted T_N_R3^−^ and T_N_R3^+^ cells (*n* = 5). Each color indicates a different donor. Data are shown as mean ± SEM. n.d., not detected. (**C**) Frequency analysis of T cell subsets in PB samples from healthy individuals (*n* = 26). Data are shown as mean ± SEM. T_SCM_ (CD45RO^−^CCR7^+^CD27^+^CD95^+^); T_CM_, central T_MEM_ (CD45RO^+^CCR7^+^); T_EM_, effector T_MEM_ (CD45RO^+^CCR7^−^); and T_TE_, terminal effector T (CD45RO^−^CCR7^−^) cells. (**D**) tSNE map displaying the surface immunophenotypes of circulating T_N_R3^−^, T_N_R3^+^, and T_MEM_ cells overlaid on the total CD8^+^ T cell population. Left, T_N_R3^−^ (red); right, T_N_R3^+^ (blue). Data were obtained using CyTOF. Individual markers are shown in (**E**). (E) Heatmap showing percent expression of the indicated markers among CD8^+^ T cell subsets identified in PB. Data were obtained using CyTOF. Subsets were defined as in (C). (**F**) Frequency analysis of T_N_R3^−^ and T_N_R3^+^ cells among total CD8^+^ T cells isolated from human tonsils (*n* = 5), spleen (*n* = 3), liver (*n* = 3), gut (*n* = 6), skin (*n* = 5), and lungs (*n* = 4). Data are shown as mean ± SEM. *p < 0.05 (paired *t* test). (**G**) Percent ratio of T_N_R3^+^/T_N_R3^−^ cells in paired LN and PB samples. Data are shown as mean ± SEM. (**H**) Frequency analysis of circulating T_N_R3^−^ and T_N_R3^+^ cells in 1938 individuals (age, 19–105 y). Red lines indicate linear regression. Effect size and *p* value are shown for each correlation.

Fluorochrome-conjugated mAbs were purchased from BD Biosciences, BioLegend, or eBioscience. All reagents were titrated before use to determine optimal concentrations (28, 29). Chemokine receptor expression was measured by incubating cells for 20 min at 37°C. Surface markers were measured by incubating cells for 20 min at room temperature. Intracellular effector molecules were revealed using a Cytofix/Cytoperm Kit (BD Biosciences). Dead cells were eliminated from the analysis using Zombie Aqua (BioLegend). Data were acquired using an LSRFortessa or a FACS Symphony A5 (BD Biosciences) and analyzed with FlowJo software version 9 (FlowJo). Naive CXCR3^−^ (T_N_R3^−^) and CXCR3^+^ (T_N_R3^+^) cells were flow-sorted using a FACSAria III (BD Biosciences). The gating strategy is depicted in Fig. 1A. Single-stained compensation controls were prepared using Ab-capture beads (BD Biosciences) as described previously (30).

### Age-associated changes in T_N_ cell subsets

T_N_R3^−^ and T_N_R3^+^ cells were quantified in venous blood samples obtained from a cohort of 1938 individuals comprising 815 males and 1123 females (age, 19–105 y) enrolled via the SardiNIA study (31, 32). To avoid circadian fluctuations and time-dependent artifacts, all samples were collected in heparin tubes at 8 am, and immunophenotyping was performed within 2 h at the recruitment site. CXCR3^−^ and CXCR3^+^ cells were quantified among naive-like CD8^+^ T cells, defined as CD3^+^CD4^−^CD45RA^+^CCR7^+^CD127^+^CD161^−^PD-1^−^. Data were acquired using a FACSAria III.

### Cell culture

Cells were cultured in RPMI 1640 medium supplemented with 10% FBS, 1% penicillin/streptomycin, and 2 mM l-glutamine (R10). To induce cytokine production, flow-sorted T_N_R3^−^ and T_N_R3^+^ cells were stimulated in a final volume of 200 μl with PMA (10 ng/ml; Sigma-Aldrich) and ionomycin (500 ng/ml; Sigma-Aldrich) for 6 h in the presence of GolgiPlug (1 μl/ml; BD Biosciences).

### Quantification of TCR excision circles

T_N_R3^−^, T_N_R3^+^, stem cell–like T_MEM_ (T_SCM_), and bulk CD45RO^+^ T_MEM_ cells were flow-sorted in PBS without Ca^2+^ and Mg^2+^, washed twice in the same buffer, and cryopreserved at −80°C. Thawed cells were lysed with proteinase K (100 μg/ml diluted in 10 mM Tris-HCl pH 8; 10 μl/100,000 cells; Roche). TCR rearrangement excision circles (TRECs) were measured using quantitative PCR (qPCR) as described previously (33) and normalized to the number of cells in each sample, determined via quantification of FAS.

### Quantification of gene expression via qPCR

Total RNA was purified using an RNeasy Micro Kit with DNAse (Qiagen), reverse transcribed using a High-Capacity cDNA Reverse Transcription Kit (Applied Biosystems), and analyzed using qPCR with hydrolysis probes for *CXCR3* (Hs00171041_m1). Reactions were set up using TaqMan Universal PCR Master Mix, No Amperase UNG (Roche) in MicroAmp Fast Optical 96-Well Reaction Plates (Applied Biosystems) and processed using an ABI 7900HT Sequence Detection System (Applied Biosystems). Expression levels were normalized (Δcycle threshold [Ct]) to the reference gene *B2M* (Hs00187842_m1) using the equation 2^− (Ct *CXCR3* – Ct *B2M*)^.

### HLA class I tetramers

Fluorochrome-conjugated tetrameric complexes of HLA-A*0201/CMV pp65_495–503_ NLVPMVATV (NV9), HLA-A*0201/influenza virus (Flu) matrix protein 1 (M1)_58–66_ GILGFVFTL (GL9), and HLA-A*0201/MART-1_26–35_ ELAGIGILTV (EV10) were generated and used as described previously (34, 35). Data were acquired from a per sample average of 6 × 10^6^ PBMCs.

### Enumeration of Ag-specific T_N_ cell precursors

Total CD8^+^ T cells and monocytes were isolated from PBMCs via positive selection using magnetic CD8 and CD14 MicroBeads, respectively (Miltenyi Biotec). Two subsets of CD4^−^CD19^−^CD56^−^CD8^+^CD45RA^+^CCR7^+^CD27^+^CD95^−^ naive cells were identified on the basis of CXCR3 expression among total CD8^+^ T cells and flow-sorted using a FACSAria III. CD8^+^ T_MEM_ cells were flow-sorted in parallel as controls. Flow-sorted T cells were cultured in RPMI 1640 medium supplemented with 5% human serum (Swiss Red Cross), 1% (v/v) nonessential amino acids, 1% (v/v) sodium pyruvate, 50 U/ml penicillin, 50 μg/ml streptomycin, and 2 mM l-glutamine (all from Invitrogen). Amplified libraries were generated in 96-well plates (2000 cells per well) via polyclonal stimulation with PHA (1 μg/ml; Remel) in the presence of irradiated (45 Gy) allogeneic feeder cells (2.5 × 10^4^ cells per well) and IL-2 (500 IU/ml) as described previously (36). Libraries were screened 14–21 d after stimulation by culturing thoroughly washed T cells (2.5 × 10^5^ cells per well) with autologous irradiated B cells (2.5 × 10^4^ cells per well) pulsed for 3 h with various Ags. The following Ags were used in these assays: a pool of 386 18-mer peptides spanning the entire 2004 consensus clade C HIV-1 proteome (1 μg/ml/peptide); a pool of 669 10-mer peptides spanning the Zika virus H/PF/2013 proteins Env, NS3, and NS5 (1 μg/ml/peptide); a pool of 198 8–11-mer peptides corresponding to immunogenic regions of CMV (1 μg/ml/peptide); a pool of 218 8–11-mer peptides corresponding to immunogenic regions of EBV (1 μg/ml/peptide); and a pool of 351 15-mer peptides spanning the Flu H1N1 strain A/California/07/2009 proteins hemagglutinin, M1, neuraminidase, and nucleoprotein (2 μg/ml/peptide). Proliferation was assessed on d 4 after incubation for 16 h with 1 μCi/ml [^3^H]thymidine (Perkin Elmer). Precursor frequencies were calculated based on the number of negative wells, assuming a Poisson distribution.

### Ag-specific T cell proliferation and effector functions

Flow-sorted T_N_R3^−^, T_N_R3^+^, and T_MEM_ cells from CMV-seronegative donors were labeled with CFSE and cultured at a ratio of 2:1 with irradiated autologous monocytes pulsed for 5 h with a human CMV lysate or a seasonal Flu vaccine (Influvac 2017/2018; Mylan). The respective cultures were supplemented with pooled CMV peptides (1 μg/ml/peptide) or pooled Flu M1 peptides (2 μg/ml/peptide). On d 10, cells were stimulated with PMA and ionomycin for 5 h in the presence of brefeldin A for the final 2 h (all reagents from Sigma-Aldrich). Cell viability was determined using a LIVE/DEAD Fixable Aqua Dead Cell Stain Kit (Thermo Fisher Scientific). Intracellular effector molecules were identified by flow cytometry after fixation/permeabilization with Cytofix/Cytoperm.

### TCR sequencing and data analysis

T_N_R3^−^, T_N_R3^+^, and T_MEM_ cells were flow-sorted in triplicate (300,000 cells per subset) directly into RLT buffer (1.2 ml; final dilution <20%; Qiagen). Total RNA was extracted using an RNeasy Mini Kit (Qiagen). Unique molecular identifier (UMI)-labeled 5′ RACE TRB cDNA libraries were prepared using a Human TCR Profiling Kit (MiLaboratory). All extracted RNA was used for cDNA synthesis, and all synthesized cDNA was used for PCR amplification. Libraries were prepared in parallel using the same number of PCR cycles and sequenced using a 150 + 150 bp approach on a NextSeq 500 (Illumina). Approximately 135 × 10^6^ TRB reads were obtained in total (1.5 ± 0.3 × 10^6^ reads per library), from which ∼4 × 10^6^ unique UMI-labeled TRB cDNA molecules were extracted using MIGEC (37) and MiXCR (38) software (53,000 ± 10,000 molecules per library), with the MIGEC threshold set to at least two reads per UMI. Each library contained an average of 40,000 ± 10,000 functional (in-frame with no stop codons) CDR3 nucleotide sequence variants (unique TRB clonotypes). Averaged physicochemical characteristics of the 5 aa residues located in the middle of the TRB CDR3 sequence (weighted by clonotype size) were analyzed using VDJtools software (39). These characteristics included the estimated energy of the interaction between cognate peptide and the TRB CDR3 (40), the strength of this interaction as a derivative of energy, volume, and hydrophobicity (Kidera factor 4) (41, 42). Diversity metrics were analyzed using VDJtools after normalization to 5000 randomly selected UMI-labeled TRB cDNA molecules per sample.

### DNA microarrays

Flow-sorted T_N_R3^−^, T_N_R3^+^, and T_MEM_ cells were washed twice in PBS without Ca^2+^ and Mg^2+^, resuspended in RLT buffer (Qiagen), and processed as described previously (43). Microarray probe fluorescence signals were converted to expression values using the robust multiarray average procedure in the Bioconductor Affy package (44). Log_2_ expression values for a total of 32,500 custom probe sets were calculated from background-adjusted and quantile-normalized fluorescence intensities using median polish summarization and custom chip definition files for the Affymetrix Human Transcriptome Array 2.0 based on Entrez genes (hta20_Hs_ENTREZG version 21.0.0). All data analyses were performed in R version 3.4.4. Differentially expressed genes (DEGs) were identified via paired comparisons of T_N_R3^−^ and T_N_R3^+^ cells using the limma algorithm in the same R package (45).

### Pathway analysis of microarray data

Mouse gene identifications obtained from comparisons between T_N_R3^+^ and CD5^lo^ or CD5^hi^ cells were converted into the corresponding human orthologous genes using the HUGO Gene Nomenclature Committee Database (https://www.genenames.org/cgi-bin/hcop). Pathway analysis was performed using gene set enrichment analysis (GSEA) software (http://software.broadinstitute.org/gsea/msigdb/) and gene sets from the Molecular Signatures Database (version 6.2). Specific gene sets included the c2 (c2.cp.reactome.v6.2) and immunological signatures collections (c7.all.v6.2). GSEA was applied to log_2_-transformed expression data obtained from T_N_R3^−^ and T_N_R3^+^ cells. Gene sets were considered significantly enriched at false discovery rate (FDR) values ≤ 0.05 using Signal2Noise as a metric across 1000 permutations.

### Mouse T_N_ cell sorting and RNA sequencing

Spleens were obtained from 12-wk-old male C57BL/6 mice (Charles River Laboratories) and mechanically smashed through a cell strainer with a pore size of 40 μm. Splenocytes were enriched for CD8^+^ T cells using a MojoSort Mouse CD8^+^ T Cell Isolation Kit (BioLegend). Flow-sorted cells were lysed in RLT buffer (50 μl; Qiagen) containing RNAse Inhibitor (1 μl; Qiagen). Total RNA was extracted using an RNeasy Micro Kit (Qiagen). RNA sequencing (RNAseq) libraries were prepared using a SMART-Seq v4 Ultra Low Input RNA Kit for Sequencing (Takara Clontech). Libraries were pooled at equimolar concentrations and sequenced on a NextSeq 500 (Illumina). At least 20 × 10^6^ single-end reads (75 bp) were generated per sample.

### RNAseq data analysis

Raw sequence data were quality controlled using FastQC (http://www.bioinformatics.babraham.ac.uk/projects/fastqc). Single-end reads (75 bp) were then aligned to the *Mus musculus* reference genome (Ensembl assembly GRCm38) using STAR (version 2.5.1b) (46). Alignments were performed using default parameters. Reads associated with annotated genes were counted using the HTSeq and “*-quantmode TranscriptomeSAM GeneCounts*” options. Differential gene expression between mouse T_N_ subsets was assessed using the edgeR package (version 3.22) (47). Benjamini-Hochberg correction was applied to estimate the FDR.

### *t*-distributed stochastic neighbor embedding analysis of high-dimensional CyTOF data

Public CyTOF data obtained from tonsils, spleen, liver, gut, skin, and lungs (27) were downloaded from https://flowrepository.org/. Debarcoded sample files were imported into FlowJo (version 9) and concatenated into a single.fcs file (∼2500 events per sample), which was then subjected to *t*-distributed stochastic neighbor embedding (tSNE) analysis (Barnes-Hut implementation) using the following parameters: iterations, 1000; perplexity, 40; initialization, deterministic; θ, 0.5; and η, 200. All markers listed in Fig. 1E were included in the analysis, except CXCR3.

### Statistics

Statistical analyses were performed using GraphPad Prism version 7 or R software version 3.4.4. Significance was assigned at *p* < 0.05 unless stated otherwise. Specific tests are indicated in the figure legends for each comparison.

### Data availability

Raw microarray and RNAseq data are available via the Gene Expression Omnibus (https://www.ncbi.nlm.nih.gov/geo/) under accession number GSE125102. Demultiplexed fastq TCR sequencing data are available via the ArrayExpress Database at The European Molecular Biology Laboratory–European Bioinformatics Institute (www.ebi.ac.uk/arrayexpress) under accession number E-MTAB-7638.

## Results

### CXCR3 identifies two subsets of T_N_ cells in humans

A previous flow cytometric analysis of human T_N_ cell populations (9), identified using stringent phenotypic criteria (CD45RO^−^CCR7^+^CD27^+^CD95^−^) to exclude memory contaminants (48, 49), demonstrated that CXCR3 was not uniformly expressed by CD8^+^ T_N_ cells. Instead, there was a clear bimodal distribution, which distinguished CXCR3^−^ (T_N_R3^−^) and CXCR3^+^ (T_N_R3^+^) cells (Fig. 1A). To confirm this finding, we flow-sorted T_N_R3^−^ and T_N_R3^+^ cells and evaluated *CXCR3* mRNA expression via qPCR. In accordance with the protein data, *CXCR3* mRNA was readily detected in T_N_R3^+^ cells, but was uncommon in T_N_R3^−^ cells (Fig. 1B). CXCR3 protein expression was detected at equivalent levels on the surface of CD8^+^ T_N_ cells with two different anti-CXCR3 mAbs (Supplemental Fig. 1A). However, a partial loss of CXCR3 expression was observed after cryopreservation (Supplemental Fig. 1B), as noted previously (50). A vast majority of our assays were therefore performed using freshly isolated PBMCs.

We then quantified T_N_ and T_MEM_ cell subsets in the PB of healthy individuals and found that T_N_R3^+^ cells were ∼3-fold more abundant than T_N_R3^−^ cells under physiological conditions (Fig. 1C). To gain further insights into the surface phenotype of these two subsets, we took advantage of a publicly available CyTOF data set reported by Wong et al. (27) who investigated the surface proteome of CD8^+^ T cells from various human tissues. Dimensionality reduction via tSNE revealed that T_N_R3^−^ and T_N_R3^+^ cells from PB (Fig. 1D) and tissues (Supplemental Fig. 1C) mapped to similar regions of the plot, indicating a common phenotype, whereas both subsets were distinct from conventional CD45RO^+^ T_MEM_ cells. A simultaneous analysis of surface markers involved in differentiation, homing, and activation/costimulation further revealed that T_N_R3^−^ and T_N_R3^+^ cells shared phenotypic traits of T_N_ cells, including the presence of CD45RA, CD27, CD62L, and CD127, and the absence of molecules such as CD49a, CD49d, CD57, CD95, CCR5, CLA, and PD-1 (Fig. 1E, Supplemental Fig. 1D) (11).

T_N_ cells preferentially migrate to secondary lymphoid organs rather than mucosal tissues (51, 52). In line with this general dichotomy, T_N_R3^−^ and T_N_R3^+^ cells were relatively abundant in human tonsils, less so in spleen and liver, and virtually undetectable in gut, skin, and lungs (Fig. 1F). On the basis of these data, it seems unlikely that CXCR3 regulates T_N_ cell trafficking under physiological conditions. Moreover, we found very similar frequencies of T_N_R3^−^ and T_N_R3^+^ cells in paired LN and PB samples (Fig. 1G), and CD8^+^ T_N_ cells almost invariably lacked the tissue-residency markers CD69 and CD103, irrespective of anatomical localization and expression of CXCR3 (Supplemental Fig. 1D).

T_N_ cells become less frequent with age (53). To investigate the impact of aging on T_N_ cell subsets, we analyzed PB samples obtained from a previously reported cohort of 1938 healthy individuals, spanning an age range from 19 to 105 y (31, 32). We found that T_N_R3^−^ and T_N_R3^+^ cells declined with age, but at slightly different rates, such that T_N_R3^+^ cells became progressively more common in the CD8^+^ T_N_ cell pool (Fig. 1H).

### True naivety of T_N_R3^+^ cells

Previous work identified CXCR3^+^ T_N_-like cells in the CD4^+^ lineage as memory precursors of Th1 cells (54). A more recent study further suggested that CXCR3^+^ T_N_-like cells in the CD8^+^ lineage were young T_MEM_ cells (55). We therefore performed a number of different assays to characterize the naive and memory properties of T_N_R3^−^ and T_N_R3^+^ cells.

**FIGURE 2.**
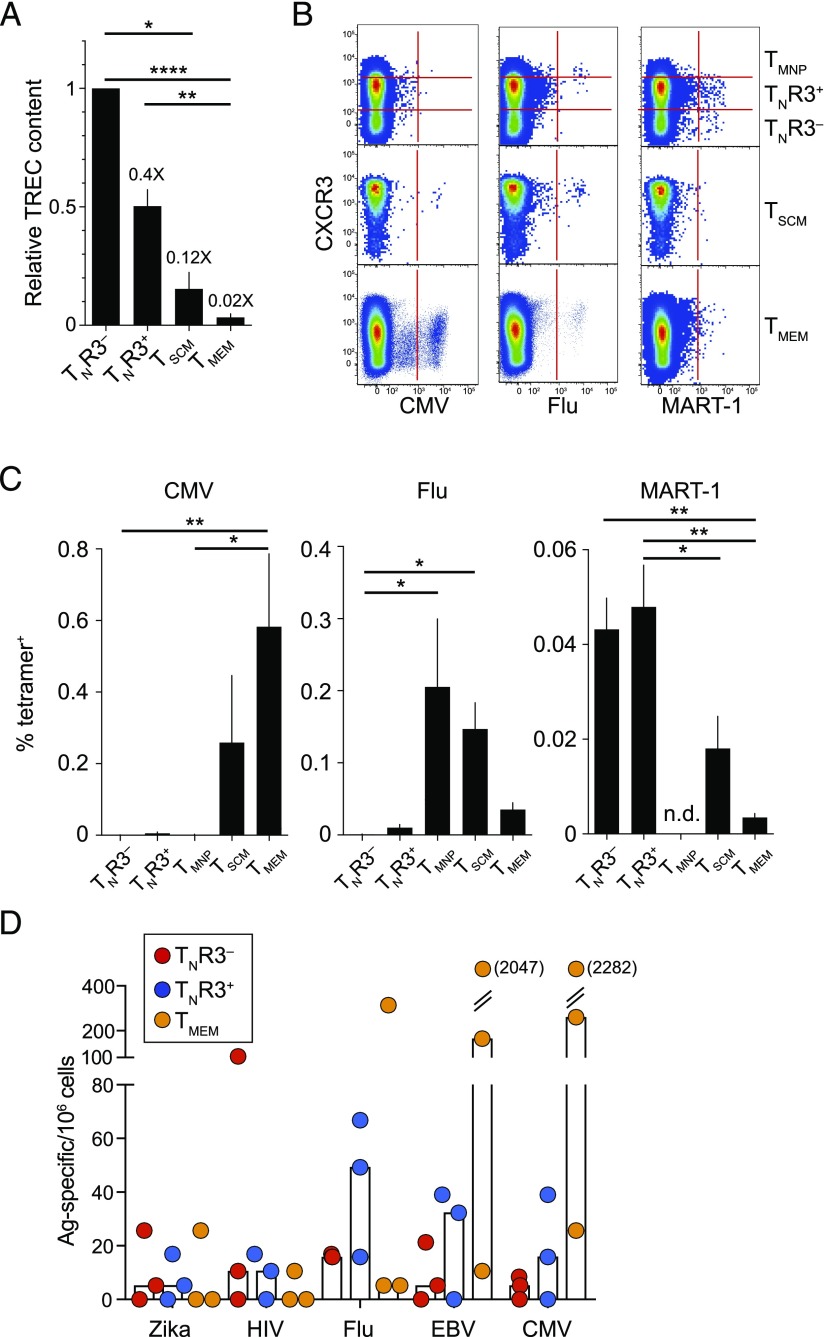
True naivety of T_N_R3^+^ cells. (**A**) TREC copies relative to T_N_R3^−^ cells in CD8^+^ T cell subsets isolated from PB (T_N_R3^−^, T_N_R3^+^, and T_MEM_ cells, *n* = 10; T_SCM_ cells, *n* = 4). Numbers indicate fold change relative to T_N_R3^−^ cells. Data are shown as mean ± SEM. *p < 0.05, **p < 0.01, ****p < 0.0001 (nonparametric ANOVA with Dunn posttest). (**B**) Representative flow cytometric analysis showing NV9 (CMV), GL9 (Flu), and EV10 (MART-1) tetramer^+^ events versus CXCR3 expression among CD8^+^ T cell subsets in PB. Plots on the top row show CD45RO^−^CCR7^+^CD27^+^CD95^−^ T_N_ cells subgated as T_N_R3^−^, T_N_R3^+^, and T_MNP_ cells. (**C**) Frequency analysis of tetramer^+^ events as shown in (B) (CMV, *n* = 6; Flu, *n* = 7; MART-1, *n* = 8). Data are shown as mean ± SEM. n.d., not detected. *p < 0.05, **p < 0.01 (nonparametric ANOVA with Dunn posttest). (**D**) Frequency analysis of Ag-specific CD8^+^ T cell precursors among T_N_R3^−^, T_N_R3^+^, and T_MEM_ cells. Bars indicate median values. Each dot represents one donor (*n* = 3).

The replicative history of T cell populations can be assessed by measuring TRECs, which are progressively diluted upon cell division (56). In ex vivo assays, we found that TRECs were ∼2-fold more common in T_N_R3^−^ cells compared with T_N_R3^+^ cells, ∼4-fold more common in T_N_R3^−^ cells compared with T_SCM_ cells (9), and ∼25-fold more common in T_N_R3^−^ cells compared with T_MEM_ cells (Fig. 2A). T_N_R3^+^ cells therefore underwent on average one additional round of division in vivo relative to T_N_R3^−^ cells, suggesting a link between homeostatic proliferation and the acquisition of CXCR3.

Truly naive T cell populations lack clonal expansions specific for exogenous Ags, but occasionally harbor large numbers of precursors specific for certain self-derived Ags (57). In line with these predictions, HLA-A*0201–restricted CD8^+^ T cells specific for immunodominant epitopes derived from CMV and Flu were uncommon in the T_N_R3^−^ and T_N_R3^+^ cell subsets, but abundant in the T_SCM_ and T_MEM_ cell subsets (9), whereas HLA-A*0201–restricted CD8^+^ T cells specific for an immunodominant epitope derived from MART-1 were common in T_N_R3^−^ and T_N_R3^+^ cell subsets (Fig. 2B, 2C). A recent study identified a rare population of memory cells with a T_N_-like phenotype (T_MNP_) that expressed high levels of CD49d and CXCR3 and rapidly produced IFN-γ in response to stimulation with PMA and ionomycin (58). CD8^+^ T cells specific for epitopes derived from persistent viruses, such as CMV and EBV, but not acute viruses, such as Flu, were detected in the T_MNP_ cell pool (58). In contrast, we detected Flu-specific CD8^+^ T cells, but not CMV-specific CD8^+^ T cells, in the T_MNP_ cell subset, which comprised ∼0.5% of the T_N_R3^+^ cell population (Fig. 2B, 2C).

Our findings with the MART-1–derived epitope suggested that Ag-specific precursors were not compartmentalized to particular subsets of T_N_ cells. To confirm this inference at the level of exogenous Ags, we screened amplified libraries of T_N_R3^−^, T_N_R3^+^, and T_MEM_ cells with peptide-pulsed APCs (36). Similar frequencies of CD8^+^ T cells specific for previously unencountered (HIV-1 and Zika virus) and more prevalent viruses (CMV, EBV, and Flu) were detected in the T_N_R3^−^ and T_N_R3^+^ cell pools (Fig. 2D). An exception was noted in one donor, who harbored remarkably high frequencies of HIV-1–specific T_N_R3^−^ cells, but not HIV-1–specific T_N_R3^+^ cells, potentially indicating degenerate recognition by cross-reactive TCRs (59). As expected, CD8^+^ T cells specific for previously unencountered viruses were largely undetectable in the T_MEM_ cell pool, whereas CD8^+^ T cells specific for more prevalent viruses were common in the T_MEM_ cell pool (Fig. 2D).

### T_N_R3^+^ cells are biased toward effector differentiation

**FIGURE 3.**
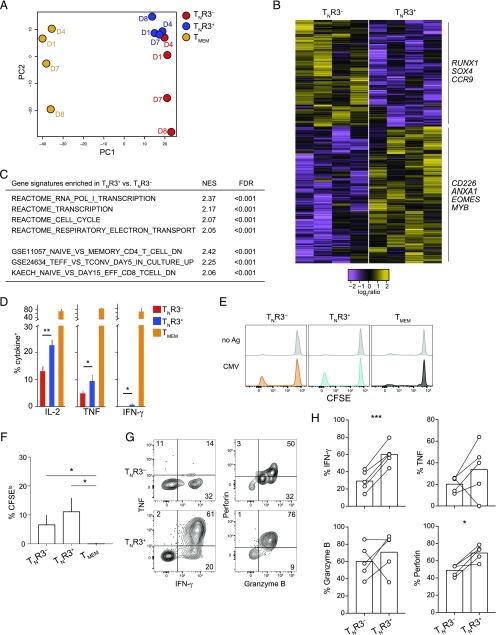
T_N_R3^+^ cells are biased toward effector differentiation. (**A**) Principal component analysis based on the expression levels of genes with coefficients of variation larger than the 90th percentile of the coefficients of variation in the entire data set, determined via microarray analysis. Labels indicate donors (*n* = 4). (**B**) Heatmap showing DEGs (*p* < 0.01) in T_N_R3^−^ versus T_N_R3^+^ cells (*n* = 4). The most relevant genes associated with immune functions are listed. (**C**) Normalized enrichment score (NES) and FDR for each gene signature enriched in T_N_R3^+^ versus T_N_R3^−^ cells, determined via GSEA. (**D**) Frequency analysis of cytokine production by T_N_R3^−^, T_N_R3^+^, and T_MEM_ cells after stimulation with PMA and ionomycin (T_N_R3^−^ and T_N_R3^+^, *n* = 6; T_MEM_ cells, *n* = 2). Data are shown as mean ± SEM. *p < 0.05, **p < 0.01 (paired *t* test). Statistics were omitted for comparisons with T_MEM_ cells. (**E**) Representative flow cytometric analysis of CFSE dilution in T_N_R3^−^, T_N_R3^+^, and T_MEM_ cells after stimulation for 10 d with autologous monocytes presenting epitopes derived from CMV. (**F**) Frequency analysis of T_N_R3^−^, T_N_R3^+^, and T_MEM_ cells that proliferated (CFSE^lo^) in response to stimulation for 10 d as in (E) (*n* = 5). Data are shown as mean ± SEM. *p < 0.05 (nonparametric ANOVA). (**G**) Representative flow cytometric analysis of effector molecules produced by CFSE^lo^ T_N_R3^−^ and CFSE^lo^ T_N_R3^+^ cells [identified as in (E)] after stimulation with PMA and ionomycin. Numbers indicate the percentage of cells in each gate. (**H**) Mean summary of data obtained as in (G). Each dot represents a different donor. *p < 0.05, ***p < 0.001 (paired *t* test).

In further experiments, we assessed the relationship between T_N_R3^−^, T_N_R3^+^, and T_MEM_ cells at the level of gene expression. Principal component analysis of the entire data set revealed that T_N_R3^−^ and T_N_R3^+^ cells were largely distinct from conventional T_MEM_ cells (Fig. 3A). Paired analysis of samples isolated from individual donors (*n* = 4) identified 345 genes that were differentially expressed (*p* < 0.01) between T_N_R3^−^ and T_N_R3^+^ cells (Supplemental Table I). The effector/memory–related transcripts *EOMES*, *MYB*, and *ANXA1* (60), and the costimulatory receptor *CD226*, which encodes DNAX accessory molecule-1 (DNAM-1), were preferentially expressed in T_N_R3^+^ cells (Fig. 3B). A total of 2567 DEGs, including *BHLHE40*, a transcription factor associated with effector differentiation, and *NT5E*, which encodes CD73, a surface enzyme involved in the generation of adenosine, were identified using a less stringent cut-off (*p* < 0.05; Supplemental Table I). Differential expression of CD73 and CD226 was further confirmed at the protein level via flow cytometry (Supplemental Fig. 1E). In contrast, transcription factors associated with the regulation of thymocyte differentiation, such as *RUNX1*, *SOX4*, and *IKZF1*, were overexpressed in T_N_R3^−^ cells (Fig. 3B, Supplemental Table I).

To capture global trends in gene expression, we performed GSEA. This approach revealed that gene sets involved in the cell cycle, transcriptional activity, and the respiratory electron transport chain (REACTOME Database), as well as transcripts associated with effector and memory activity (Immunological Signatures Database), were strongly enriched in T_N_R3^+^ versus T_N_R3^−^ cells (FDR < 0.001; Fig. 3C). These findings suggested that T_N_R3^+^ cells were better poised to differentiate and acquire effector functionality compared with T_N_R3^−^ cells. To test this hypothesis, we stimulated flow-sorted T_N_R3^−^, T_N_R3^+^, and T_MEM_ cells directly ex vivo with PMA and ionomycin. Twice as many T_N_R3^+^ cells produced IL-2, potentially reflecting decreased expression of *IKZF1*, an inhibitor of IL-2 production in CD8^+^ T_N_ cells (61), and TNF compared with T_N_R3^−^ cells (Fig. 3D). In line with their naive status, however, both T_N_R3^−^ and T_N_R3^+^ cells largely failed to produce IFN-γ, unlike T_MEM_ cells (Fig. 3D). The corresponding subsets were also flow-sorted from CMV-seronegative donors and cultured for 10 d with autologous monocytes presenting epitopes derived from CMV. Ag-driven proliferation was observed in the T_N_R3^−^ and T_N_R3^+^ cell subsets, but not in the T_MEM_ cell subset (Fig. 3E, 3F). Importantly, CMV-specific T_N_R3^+^ cells that underwent proliferation, assessed via serial dilution of CFSE, produced more IFN-γ and perforin and tended to produce more TNF compared with T_N_R3^−^ cells after stimulation with PMA and ionomycin on d 10 (Fig. 3G, 3H).

### T_N_R3^−^ and T_N_R3^+^ cells express qualitatively distinct TCRs

**FIGURE 4.**
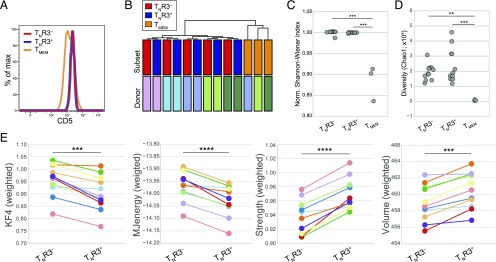
T_N_R3^−^ and T_N_R3^+^ cells express qualitatively distinct TCRs. (**A**) Representative flow cytometric analysis of CD5 expression on the surface of human T_N_R3^−^, T_N_R3^+^, and T_MEM_ cells. Similar data were obtained from three other donors. (**B**) Cluster analysis of TRBV-TRBJ use among T_N_R3^−^, T_N_R3^+^, and T_MEM_ cells (T_N_R3^−^ and T_N_R3^+^, *n* = 5; T_MEM_ cells, *n* = 3). (**C**) Normalized Shannon-Wiener diversity index and (**D**) Chao1 diversity index calculated for 5000 unique UMI-labeled TRB CDR3 molecules from two independent experiments (*n* = 11). ***p < 0.001 (Tukey range test). (**E**) Averaged (weighted per clonal size) Kidera factor 4 (KF4), Miyazawa-Jernigan energy (MJenergy), strength, and volume indices for the 5 aa residues located the middle of the TRB CDR3 sequences extracted from the T_N_R3^−^ and T_N_R3^+^ cell repertoires [details as in (D)]. **p < 0.01, ***p < 0.001, ****p < 0.0001 (paired *t* test).

In mice, enhanced TCR reactivity against self-derived Ags correlates with surface expression of CD5 and determines the efficiency of T_N_ cell recruitment in response to foreign Ags (20–22). We found that surface levels of CD5 were comparable between T_N_R3^−^ and T_N_R3^+^ cells (Fig. 4A). However, the strength of TCR interactions with pMHCI molecules can also be inferred from the physicochemical properties of TRB CDR3 sequences (23, 40, 62, 63), as was recently proposed for human and mouse regulatory T cells (64–66) and CD4^+^ T cells (67). We therefore used a high-throughput approach to sequence the TRB repertoires of flow-sorted T_N_R3^−^, T_N_R3^+^, and T_MEM_ cells. Importantly, T_N_R3^−^ and T_N_R3^+^ cells from individual donors exhibited similar patterns of TRBV-TRBJ use (Jenson-Shannon divergence analysis), indicating a close relationship, whereas distinct patterns of TRBV-TRBJ use were observed in the corresponding T_MEM_ cell subsets (Fig. 4B). Repertoire diversity was comparably high in the T_N_R3^−^ and T_N_R3^+^ cell subsets, as expected for truly naive populations, but much lower in the corresponding T_MEM_ cell subsets, quantified using normalized Shannon-Weiner and Chao1 metrics (Fig. 4C, 4D). We then determined the averaged physicochemical properties of the 5 aa residues located in the middle of each TRB CDR3 sequence, which dominate interface contacts with the peptide component of pMHCI molecules (62). At the population level, increased hydrophobicity (lower Kidera factor 4), lower energy (40, 63), and higher strength and volume indices, calculated using VDJtools (39), were observed for TRB CDR3 sequences in the T_N_R3^+^ cell pool compared with TRB CDR3 sequences in the T_N_R3^−^ cell pool (Fig. 4E).

### T_N_R3^+^ cells are transcriptionally equivalent in humans and mice

**FIGURE 5.**
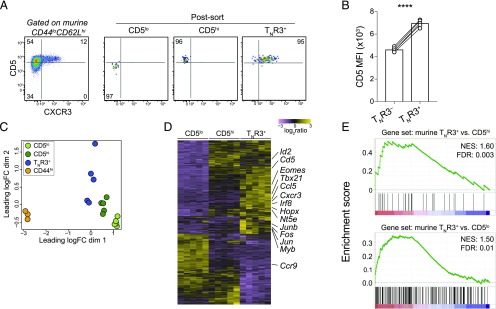
T_N_R3^+^ cells are transcriptionally equivalent in humans and mice. (**A**) Left: representative flow cytometric analysis of CD5 and CXCR3 expression on the surface of murine CD8^+^ T_N_ cells (CD44^lo^CD62L^hi^). Right: postsort purity analysis of murine CD5^lo^, CD5^hi^, and T_N_R3^+^ cells. (**B**) Mean fluorescence intensity (MFI) of CD5 expression on the surface of murine T_N_R3^−^ and T_N_R3^+^ cells, gated as CD44^lo^CD62L^hi^CXCR3^−^ and CD44^lo^CD62L^hi^CXCR3^+^, respectively (*n* = 5). ****p < 0.0001 (paired *t* test). (**C**) Biological coefficient of variation (BCV) plot derived from RNAseq profiles of murine CD5^lo^, CD5^hi^, and T_N_R3^+^ cells isolated as in (A) (*n* = 5). Bulk memory CD8^+^ T cells are shown as CD44^hi^ (*n* = 3). (**D**) Heatmap showing DEGs (FDR < 0.0001) among CD5^lo^, CD5^hi^, and T_N_R3^+^ cells isolated as in (A). The most relevant genes associated with immune functions are listed. (**E**) GSEA plots showing murine T_N_R3**^+^** versus CD5^hi^ (top) and murine T_N_R3**^+^** versus CD5^lo^ gene sets (bottom) that were significantly enriched in human T_N_R3**^+^** versus T_N_R3^−^ cells (Fig. 3C).

To corroborate these findings, which suggested that TCRs with higher intrinsic affinities for self-derived Ags were more prevalent in the T_N_R3^+^ cell pool compared with the T_N_R3^−^ cell pool, we extended our analysis to the murine CD8^+^ T_N_ compartment (defined as CD44^lo^CD62L^hi^). Murine T_N_R3^+^ cells were contained almost exclusively within the CD5^hi^ fraction (Fig. 5A, 5B). These cells were shown previously to respond more vigorously to foreign Ags compared with CD5^lo^ and CD5^hi^ T_N_ cells (22). To characterize murine T_N_R3^+^ cells in more detail, we flow-sorted CD5^lo^CXCR3^−^ (CD5^lo^), CD5^hi^CXCR3^−^ (CD5^hi^), and CD5^hi^CXCR3^+^ (T_N_R3^+^) cells from the splenic CD44^lo^CD62L^hi^ T_N_ cell pool, together with CD44^hi^ T_MEM_ cells, and defined the transcriptional profile of each subset using RNAseq. All three T_N_ cell subsets were clearly distinct at the transcriptional level compared with the conventional T_MEM_ cell subset, based on the biological coefficient of variation (Fig. 5C). However, we also identified 636 DEGs (FDR < 0.001) among the T_N_ cell subsets (Fig. 5D, Supplemental Table II). In addition to *Cd5* and *Cxcr3*, murine T_N_R3^+^ cells overexpressed genes associated with effector differentiation, including *Tbx21*, *Ccl5, Irf8, Hopx, Junb, Fos*, and *Jun* (Fig. 5D, Supplemental Table II), and with less stringent criteria (FDR < 0.05), underexpressed genes associated with a naive phenotype, including *Lef1* and *Ccr7*, compared with both CD5^lo^ and CD5^hi^ T_N_ cells (Supplemental Table II). Importantly, DEGs identified in the corresponding human T_N_ cell subsets, such as *Ccr9*, *Eomes*, *Nt5e*, *Myb*, *Sox4*, and *Ikzf1*, were also differentially expressed among murine T_N_ cell subsets (Fig. 5D, Supplemental Table II). In accordance with these data, genes upregulated in murine T_N_R3^+^ versus CD5^lo^ (*n* = 221; FDR < 0.0001) and murine T_N_R3^+^ versus CD5^hi^ cells (*n* = 37; FDR < 0.0001) were also preferentially enriched in human T_N_R3^+^ versus T_N_R3^−^ cells, indicating close transcriptional parallels between T_N_R3^+^ cells in different species (Figs. 3C, 5E).

## Discussion

It has become apparent in recent years that the classically defined T_N_ cell pool incorporates subpopulations of cells with memory-like properties, including T_SCM_ cells (9, 51) and T_MNP_ cells (58). In this study, we found that truly naive T cells can also exhibit distinct characteristics, both in humans and in mice. Specifically, we identified two discrete subsets of CD8^+^ T_N_ cells in each species, defined by the absence or presence of the chemokine receptor CXCR3. In humans, T_N_R3^+^ cells more frequently produced IL-2 and TNF in response to nonspecific activation directly ex vivo, differentiated more readily to acquire various effector functions in vitro after stimulation with cognate Ag, and overexpressed a distinct array of genes compared with T_N_R3^−^ cells. Repertoire analysis further indicated that T_N_R3^+^ cells expressed TCRs with enhanced Ag sensitivity, despite comparably high levels of diversity in the T_N_R3^−^ and T_N_R3^+^ cell pools. Moreover, human T_N_R3^+^ cells were phenotypically and transcriptionally equivalent to murine T_N_R3^+^ cells, which expressed high levels of CD5. It is notable in this regard that CXCR3 has also been shown to demarcate functionally superior CD8^+^ T cells that respond to innate signals in the murine CD44^hi^ memory compartment (68, 69).

In mice, the ability of CD8^+^ T_N_ cells to respond to infectious agents has been shown to correlate with TCR sensitivity for self-derived Ags (22), which can be assessed by measuring surface expression of CD5 (20–23). Accordingly, genes associated with the cell cycle and effector differentiation, including *Tbx21* and *Eomes*, were upregulated in CD5^hi^ T_N_ cells compared with CD5^lo^ T_N_ cells (22). Some murine CD5^hi^ T_N_ cells also expressed CXCR3 (22). We found that these murine T_N_R3^+^ cells overexpressed several transcripts associated with effector differentiation compared with both CD5^lo^ and CD5^hi^ cells, closely mirroring the transcriptional identity of human T_N_R3^+^ cells. It remains unclear to what extent CD5 can be used to identify functionally distinct subsets of human T_N_ cells (24, 25). However, T_N_ cells with a proclivity for effector differentiation were clearly defined in our data set on the basis of CXCR3 expression, thereby providing a unique identifier for the isolation of truly naive precursors with functional properties that may be useful in the context of various immunotherapies.

Recent data have shown that memory-like CD8^+^ T cells are retained in the skin and thymus of nonimmunized mice via a CXCR3-dependent mechanism (70). We found that T_N_R3^+^ cells were virtually excluded from mucosal sites, including the skin, but were relatively abundant in PB and LNs. It also seems likely that T_N_R3^−^ and T_N_R3^+^ cells recirculate continuously between these latter two compartments, because neither subset expressed the tissue-residency markers CD69 and CD103. In line with these findings, another study reported that CXCR3-dependent signaling was undetectable in naive and memory CD8^+^ T cells directly ex vivo, but increased with stimulation via the TCR (71). Moreover, the CXCR3 ligands CXCL9, CXCL10, and CXCL11 are poorly expressed at steady-state and only become upregulated in the context of inflammation (72, 73). The functional and migratory advantages of T_N_R3^+^ cells relative to T_N_R3^−^ cells may therefore be confined to the setting of immune activation, as described previously for murine CXCR3^+^ T_CM_ cells, which localize rapidly to peripheral areas of the relevant LNs in response to challenge with previously encountered Ags (73).

TRECs were modestly diluted in T_N_R3^+^ cells compared with T_N_R3^−^ cells, indicating a slightly higher rate of homeostatic turnover in vivo, akin to that observed previously for CD5^hi^ T_N_ cells compared with CD5^lo^ T_N_ cells in lymphopenic mice (23). Homeostatic proliferation may even drive the acquisition of CXCR3 (74). In this scenario, T_N_R3^+^ cells would arise as a natural consequence of enhanced tonic signaling via qualitatively distinct TCRs with a predilection for self-derived Ags. Alternatively, T_N_R3^+^ cells may be preprogrammed during thymic development to respond more vigorously to homeostatic signals in the periphery. In line with this possibility, a recent study demonstrated that murine T_N_ cells generated early in life, which resembled T_N_R3^+^ cells at the transcriptional level, were more prone to effector differentiation than murine T_N_ cells generated later in life (75).

In summary, we have shown that humans and mice harbor at least two distinct subsets of T_N_ cells, defined by the absence or presence of CXCR3. The greater effector differentiation potential of T_N_R3^+^ cells bestows obvious kinetic advantages, enabling timely immune responses in the face of perceived threats to the host (22). The biological role of T_N_R3^−^ cells is less clear. A parallel repertoire of qualitatively distinct TCRs may nonetheless be required to cover any potential “holes” in the spectrum of adaptive immune specificities, even at the cost of a selection disadvantage in the overall T_N_ cell pool (76). On this basis, we propose that the preimmune repertoire is organized into functionally and transcriptionally discrete subsets, which fulfill different roles in the immune system, collectively ensuring balanced and comprehensive effector responses to exogenous Ags.

## Supplementary Material

Data Supplement
