# Supplementary material for: CXCR3 Identifies Human Naive CD8+ T Cells with Enhanced Effector Differentiation Potential
Source: J Immunol. 2019 Nov 18;203(12):3179–89. doi: 10.4049/jimmunol.1901072 (PMC6900484; doi:10.4049/jimmunol.1901072)
Supplement: Data Supplement [file JI_1901072.zip › JI_1901072_Supplemental_Figure_1.pdf]

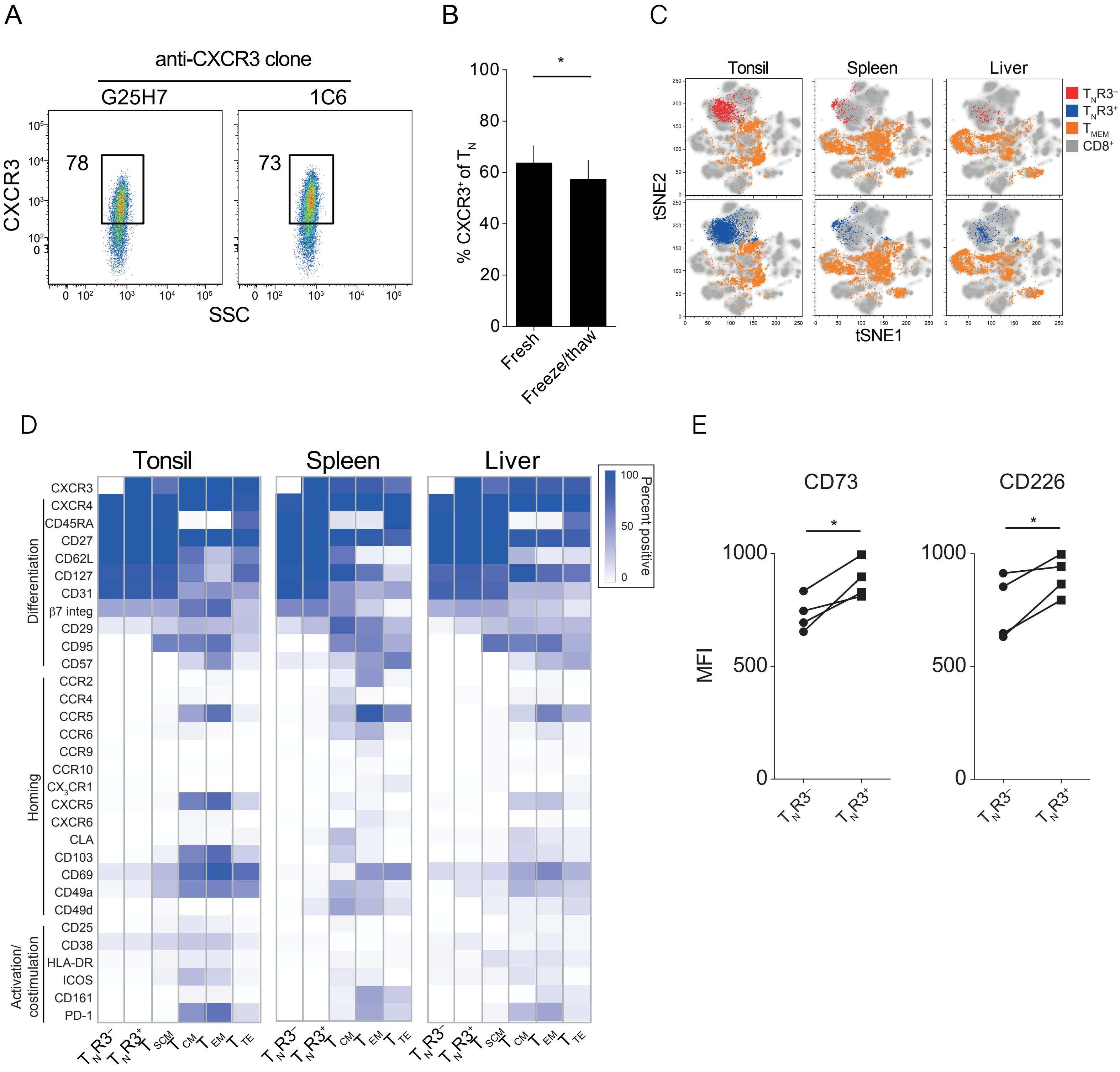

Figure S1. Identification of CXCR3⁺ human CD8⁺ T_N cells. (A) Representative flow cytometric analysis of CXCR3 expression on the surface of CD8⁺ T_N cells, detected using two different mAbs. Similar data were obtained from four other donors. **(B)** Frequency analysis of CXCR3 expression on the surface of CD8⁺ T_N cells before and after cryopreservation (n = 5). Data are shown as mean ± SEM. * $p < 0.05$ (paired t-test). **(C)** tSNE map displaying the surface immunophenotypes of T_NR3⁻, T_NR3⁺, and TMEM cells from the indicated tissues overlaid on the corresponding total CD8⁺ T cell populations. Data were obtained using CyTOF. Individual markers are shown in B. **(D)** Heatmap showing percent expression of the indicated markers among CD8⁺ T cell subsets identified in the indicated tissues. Data were obtained using CyTOF. Subsets were defined as in Figure 1C. **(E)** Mean fluorescence intensity (MFI) of CD73 and CD226 expression on the surface of T_NR3⁻ and T_NR3⁺ cells (n = 4). * $p < 0.05$ (paired t-test). Related to Figure 3.
